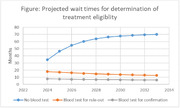# Impact of a High‐Performing Blood Test on Wait Times for Determination of Eligibility for a Disease‐Modifying Alzheimer’s Treatment in the U.S.

**DOI:** 10.1002/alz.091612

**Published:** 2025-01-09

**Authors:** Soeren Mattke, Jiahe Chen, Mark Hanson

**Affiliations:** ^1^ University of Southern California, Los Angeles, CA USA; ^2^ University Of Southern California, Los Angeles, CA USA

## Abstract

**Background:**

As disease‐modifying treatments for Alzheimer’s disease (AD) are becoming available, concerns have been raised about wait times in the patient diagnostic journey due to a limited number of AD specialists and PET scanners. A high‐performance blood test used in the in primary care settings has the potential to address this concern. We estimate the impact of such a test on wait times in the U.S.

**Method:**

We use a Markov model to predict wait times for individuals identified as eligible for AD treatment, taking into account constrained capacity for AD specialist visits and confirmatory biomarker testing. We assume that individuals would undergo a brief cognitive assessment in primary care and, if indicative of early‐stage cognitive impairment, be referred to a AD specialist under three scenarios: (1) no blood test, (2) blood test to rule out AD pathology, and (3) blood test to confirm AD pathology.

We model the U.S. population aged 55+ from 2023 to 2032, assuming that 25% of all individuals, who have never been evaluated for cognitive decline, and 5%, who were previously found to be cognitively normal, would undergo a brief cognitive test. If found to have early‐stage cognitive impairment and a blood test indicative of AD (if available), 80% would be referred to an AD specialist. The AD specialist would assess the patient and order biomarker testing for 90% of patients with confirmed early‐stage cognitive impairment. Such biomarker testing would include CSF testing (10% and not capacity constrained) and PET imaging (90%). Patients would then return to the specialist to discuss test results and a treatment indication. If the blood test were used to confirm the AD pathology, patients would skip the first specialist visit.

**Result:**

The figure shows the expected wait times for the three scenarios.

**Conclusion:**

The introduction of a blood test into the AD diagnostic pathway could reduce wait times substantially and prevent eligible patients from falling outside the treatment window. Ongoing work will assess additional scenarios and patient pathways as well as their cost implications.